# Modelling of influential parameters on a continuous evaporation process by Doehlert shells

**DOI:** 10.1155/S146392460300004X

**Published:** 2003

**Authors:** Henri Fauduet, Catherine Porte, Jean-Louis Havet, David Daguet

**Affiliations:** 1 Laboratoire de Productique Chimique—EA 21 Institut Universitaire de Technologie Université d'Orléans BP 6729 Orléans-Cedex 2 F-45067 France; 2 Laboratoire de Chimie Industrielle et Génie des Procédés—EA 21 Conservatoire National des Arts et Métiers 2 rue Conté paris F-75003 France

## Abstract

The modelling of the parameters that influence the continuous evaporation of an alcoholic extract was considered using Doehlert matrices. The work was performed with a wiped falling film evaporator that allowed us to study the influence of the pressure, temperature, feed flow and dry matter of the feed solution on the dry matter contents of the resulting concentrate, and the productivity of the process. The Doehlert shells were used to model the influential parameters. The pattern obtained from the experimental results was checked allowing for some dysfunction in the unit. The evaporator was modified and a new model applied; the experimental results were then in agreement with the equations. The model was finally determined and successfully checked in order to obtain an 8% dry matter concentrate with the best productivity; the results fit in with the industrial constraints of subsequent processes.


Journal of Automated Methods & Management in Chemistry
Vol. 25, No. 1 (January–February 2003) pp. 21–30

Taylor & Francis

Taylor & Francis Group

### Modelling of influential parameters on a continuous evaporation process by Doehlert shells

**Henri Fauduet[1]*, Catherine Porte[2], Jean-Louis Havet[2] and David Daguet[1]**

[1]*Laboratoire de Productique Chimique—EA 21, Institut Universitaire de Technologie, Université d'Orléans, BP 6729, F-45067 Orléans-Cedex 2, France*
[2] *Laboratoire de Chimie Industrielle et Génie des Procédés—EA 21, Conservatoire National des Arts et Métiers, 2 rue Conté, F-75003 Paris, France*

*The modelling of the parameters that influence the continuous evaporation of an alcoholic extract was considered using Doehlert matrices. The work was performed with a wiped falling film evaporator that allowed us to study the influence of the pressure, temperature, feed flow and dry matter of the feed solution on the dry matter contents of the resulting concentrate, and the productivity of the process. The Doehlert shells were used to model the influential parameters. The pattern obtained from the experimental results was checked allowing for some dysfunction in the unit. The evaporator was modified and a new model applied; the experimental results were then in agreement with the equations. The model was finally determined and successfully checked in order to obtain an 8% dry matter concentrate with the best productivity; the results fit in with the industrial constraints of subsequent processes.*

### Introduction

The production of high added-value compounds from vegetal substrates has again become an interesting scientific and economic operation comparing favourably with chemical synthesis. The more constraining regulations about security and environmental protection increase, the increasing costs of industrial raw materials and the decreasing prices of agricultural products give the natural processes a competitive footing with the synthetic products. Moreover, some phytomolecules, in particular optically active ones, cannot be obtained by synthetic routes. However, geneticists are often in a position to increase the capacity of a plant to produce particular molecules in the plant.

The isolation of a molecule from a plant is carried out in several steps by combining unit operations with simple chemical modifications, thus allowing the separation of a family of components from the medium. The first step usually consists of leaching the vegetal matter with a convenient solvent in previously optimized conditions to obtain both an extract and a solid residue. The dry matter (DM) content of the extract depends on the nature of the solvent and the conditions of extraction. The second step is often a partial evaporation giving a concentrate that exhibits the content level required by the subsequent steps of the process (physicochemical unit operation, chemical reaction, etc.).

The purpose of the present work was to obtain, on a pilot scale, an 8% DM concentrate from the variable solid content of a feed extract resulting from leaching, with the best productivity of evaporation. To reach these results, the building of the model for the influence of several parameters for a continuous evaporation (temperature, absolute pressure, feed flow, dry matter of feed solution) of an alcoholic extract was considered using Doehlert matrices.

### Materials and methods

### *Wiped falling film evaporator*

The evaporation was performed in a continuously wiped falling film evaporator (Luwa-type). This evaporator is particularly suitable when compared with batch evaporators for concentrating thermosensitive products since the residence time on the hot supply is relatively short (some 10 s) and depends on the viscosity of the concentrate. The operation was carried out according to the following procedure (figure 1). The feed solution (F) is dispatched to the top of the evaporator thanks to a volumetric pump (P) (Prominent Gamma/5) fitted with a counter-pressure valve. The solution enters the unit tangentially above the heated zone and is distributed evenly over the inner circumference of the body wall by the rotor. The wiping blade (S) induces the product to spiral down along the hot wall. The volatile components are rapidly evaporated co-currently with the warming fluid at a temperature measured by the TI1 probe and are then condensed in a triple coil heat exchanger (HE1). The inlet and outlet temperatures of the cooling water are measured by the TI2 and TI3 probes. Non-volatile components (concentrate) are discharged at the bottom outlet of the unit. Continuous washing by the bow waves minimizes the fouling of the thermal wall where the residue concentrates most. The concentrate (C) and evaporate (E) are continuously collected in the corresponding tanks after streaming on the respective cooling pipes (HE2 and HE3). The warming fluid, heated and regulated at a temperature indicated by the TIC probe in a thermostat (Th) ('GMC es 13 M' type with a 6 kW power supplied by Parmilleux, Vaulx-en-Velin, France), flows through the double jacket of the evaporator before being recycled in the thermostat. A vacuum is obtained by a water-sealed rotary pump and is regulated at the studied value, directly from the control cupboard, by an electro-valve (EV1) controlling an escape.

* To whom correspondence should be addressed. e-mail henri.fauduet@iut.univ-orleans.fr

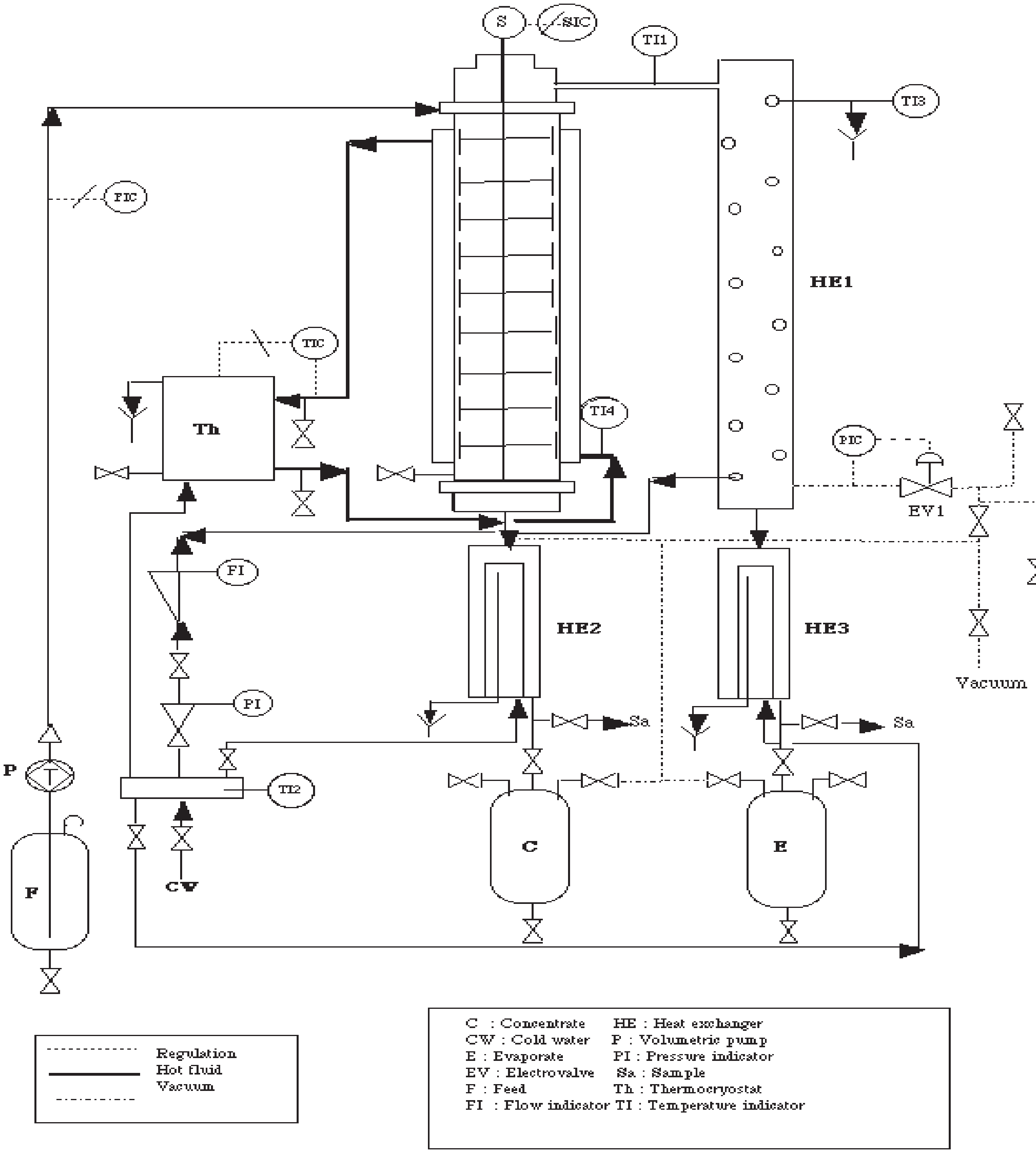

*Figure 1. Set-up of the wiped falling film evaporator.*

With this unit, designed by Pignat SA (Genas, France), it is possible to change the influential parameters within the following ranges for (1) absolute pressure (PIC): from 50 hPa to atmospheric pressure; (2) the temperature of the warming fluid (TIC): from room temperature to 120 °C; (3) feed flow (FIC): from 0 to 10 l h$^{-1}$; (4) the stirring speed of the wiping blade (SIC): from 0 to 180 rpm. The latter parameter was not studied and the speed was maintained at 120 rpm.

*Measures*

The dry matter contents of samples were determined by evaporation of solvent in a drying oven at 105 °C for at least 4 h. The flows of evaporate ($E$) and concentrate ($C$) were calculated by measuring the weight of the effluents obtained during a given time. The densities were obtained by the pycnometric method. Pressures (hPa) were absolute pressures. The working temperature was given by a probe situated at the inlet of the evaporator (TI4). For every measure the mass balance law was verified with an error inferior to 5% by carrying out the balance on the overall weights ($F = C + E$) and on the input and output dry matter ($F.w_F = C.w_C$), where $w_f$ and $w_c$ are, respectively, the mass fraction of the feed solution and the concentrate. $C.w_C$ represents also the productivity of the operation.

*Doehlert matrices*

The model was built using the Doehlert lattices because with this method only a few experiments are required for a given number of studied parameters. The Doehlert matrices offer the possibility of continuing studying the processes by adding other factors without modifying the preliminary results and also of performing a translation of the experimental area to delimit the optimum better.

A Doehlert matrix is generated from a simplex and represents the meshes of a lattice of points uniformly distributed in the space at the same distance from the centre [1, 2]. It leads to an estimate of the efficiencies of second-level polynomial models, which allows one to predict a response in every point of the studied area. A Doehlert matrix is built in two steps. The first step is generated from the initial simplex with $(k+1)$ vertices in a $k$-dimensional space. The first vertex of the simplex must be the centre of the experimental area, and the other points are the coordinates of an equilateral triangle (two factors), a tetrahedron (three factors), a hypertetrahedron (four factors), etc. The coordinates of the initial simplex, for a matrix with three factors (tetrahedron), are shown in table 1. Every simplex with $k$ variables can be deduced from the simplex immediately inferior (with $k-1$ variables) by adding a line with the coordinates given in table 2 (with $p = k-1$). So, for a four-factor matrix ($k = 4$), the values of $X_1(p = 3)$, $X_2(p = 2)$ and $X_3(p = 1)$ of experiment 5 are given by relation I, and $X_4$ by relation II (table 2). The points of the initial simplex for a four-factor Doehlert matrix are given in the first five lines of table 3. The additional points are then obtained by subtracting the coordinates two by two from the vertex of the initial simplex (table 3). The principle for building a matrix with two or three factors is shown in figure 2: points A (0; 0), B (1; 0) and C (0.5; 0.866) are the coordinates of an equilateral triangle (regular simplex in a two-dimensional space figure 2A). The subtraction of points, the one from the other (E = A − B; F = A − C; G = B − C, H = C − B), leads to a regular hexagon with a centre point (figure 2B). The tetrahedron, obtained in a three-dimensional space, is shown in figure 2C.

*Table 1. First points of a three-factor Doehlert matrix (simplex).*

| | Variables | | |
|---|---|---|---|
| Experiment | $X_1$ | $X_2$ | $X_3$ |
| 1 | 0 | 0 | 0 |
| 2 | 1 | 0 | 0 |
| 3 | 0.5 | 0.866 | 0 |
| 4 | 0.5 | 0.289 | 0.816 |

Doehlert matrices are rotatable ($d_i$ is constant in all the settings of the experimental variables, $x_i$ is at the same distance $r$ from the centre point of the design) but lead to a high variance as a result of the small number of experiments required in comparison with second-order experimental designs. For example, the study of four factors requires 21 experiments with a Doehlert matrix and at least 25 experiments with a central composite design. Doehlert matrices present several specifications.

(1) The experimental results obtained when using a Doehlert matrix lead to the estimation of several coefficients of a second-order polynomial model: one $b_0$ coefficient, $kb_i$ first-order coefficients, $kb_i^2$ second-order coefficients and $[k(k-1)/2]b_{ij}$ interaction coefficients.

(2) The number of experiments is not high. The minimal number of points is given by the relation $N = k^2 + k + 1$, where $k$ is the number of factors studied, whereas a central composite design requires a minimal number of experiments given by $N = 2^k + 2k + N_0$, where $N_0$ is the number of experiments performed at the centre of the area. However, Doehlert matrices involve only one point at the centre but several experiments are recommended at this point.

(3) Contrary to what occurs with classical experimental designs, the number of levels studied for each factor is not equal: five for the first, three for the last one and seven for the others for a four-variable matrix (table 3). In view of this, it will be possible to assign the most sensitive parameters to the intermediary variables $X_2$ and $X_3$ in a four-factor matrix. In the same way, when the number of levels of a parameter need to be restricted, it is possible to allocate this factor to the last variable.

(4) Doehlert matrices offer the possibility of studying one (or several) additional factor(s) without any change in the already performed experiments. The changeover from three to four factors implies only eight new experiments while keeping the 13 original points. However, its feasibility supposes that the results remain homogeneous in time with experiments.

*Table 2. Calculation of the additional points of a Doehlert matrix.*

| Variables | $X_{(k-p)}$ | ··· | $X_{(k-2)}$ | $X_{(k-1)}$ | $X_k$ |
|---|---|---|---|---|---|
| $(k+1)$th experiment | $\frac{1}{\sqrt{2[(k+1)-p](k-p)}}$ (I) | ··· | $\frac{1}{\sqrt{2(k-1)(k-2)}}$ | $\frac{1}{\sqrt{2k(k-1)}}$ | $\sqrt{\frac{(k+1)}{2k}}$ (II) |

*Table 3. Four-factor Doehlert matrix.*

| | Variables | | | |
|---|---|---|---|---|
| Experiment | $X_1$ | $X_2$ | $X_3$ | $X_4$ |
| 1 | 0 | 0 | 0 | 0 |
| 2 | 1 | 0 | 0 | 0 |
| 3 | 0.5 | 0.866 | 0 | 0 |
| 4 | 0.5 | 0.289 | 0.816 | 0 |
| 5 | 0.5 | 0.289 | 0.204 | 0.791 |
| 6 (1–2) | −1.0 | 0 | 0 | 0 |
| 7 (1–3) | −0.5 | −0.866 | 0 | 0 |
| 8 (1–4) | −0.5 | −0.289 | −0.816 | 0 |
| 9 (1–5) | −0.5 | −0.289 | −0.204 | −0.791 |
| 10 (2–3) | 0.5 | −0.866 | 0 | 0 |
| 11 (2–4) | 0.5 | −0.289 | −0.816 | 0 |
| 12 (2–5) | 0.5 | −0.289 | −0.204 | −0.791 |
| 13 (3–2) | −0.5 | 0.866 | 0 | 0 |
| 14 (3–4) | 0 | 0.577 | −0.816 | 0 |
| 15 (3–5) | 0 | 0.577 | −0.204 | −0.791 |
| 16 (4–2) | −0.5 | 0.289 | 0.816 | 0 |
| 17 (4–3) | 0 | −0.577 | 0.816 | 0 |
| 18 (4–5) | 0 | 0 | 0.612 | −0.791 |
| 19 (5–2) | −0.5 | 0.289 | 0.204 | 0.791 |
| 20 (5–3) | 0 | −0.577 | 0.204 | 0.791 |
| 21 (5–4) | 0 | 0 | −0.612 | 0.791 |
| Number of levels | 5 | 7 | 7 | 3 |

(5) It is also possible to perform a translation of the initial matrix while keeping several points of the original matrix (only three new experiments instead of seven for four factors, seven new points instead of 11 for three factors, with only one point at the centre, etc.) when the results allow to research the optimum in a related area.

### Results and discussion

### *Three-factor matrix*

The initial aim of the study was to link the influence of absolute pressure, feed flow and temperature of the heat-exchange fluid on the concentration of an alcoholic extract. The crude extract was obtained after leaching a plant with ethanol using a continuous screw-conveyor extractor ('De Smet' type). This first series of experiments on concentration was carried out on a 1.76% DM extract obtained from the latter unit. The actual variables were calculated from the following relations, where $X_1$, $X_2$ and $X_3$ are the pressure, the volume feed flow of the solution and the temperature expressed in coded units, according to Box's notation [3]: (1) $p = 150 + 29X_1$; (2) $q_v = 7 + 1.73X_2$; and (3) $T = 55 + 18.4X_3$. This experimental area was chosen because the farthest values of these quantities are compatible with the technological capability of the unit and the physicochemical properties of the extract.

The results are shown in table 4. The matrix calculation, carried out according to the least-squares method, gives the estimated pattern represented by the following equations for dry matter content (equation 1) and for productivity (equation 2) (terms in italics are not significant):

$$Y_1 = 2.55 - 0.49X_1 - 0.86X_2 + 2.62X_3 + \mathit{0.16X_1^2} + \mathit{0.16X_2^2} + 2.03X_3^2 + \mathit{0.20X_1X_2} - \mathit{0.82X_1X_3} - 2.51X_2X_3 \qquad (1)$$

$$Y_2 = 0.1049 + \mathit{0.0002X_1} + 0.0257X_2 + \mathit{0.0004X_3} - \mathit{0.0010X_1^2} - \mathit{0.0011X_2^2} + \mathit{0.0008X_3^2} - \mathit{0.0004X_1X_2} - \mathit{0.0019X_1X_3} + \mathit{0.0004X_2X_3}. \qquad (2)$$

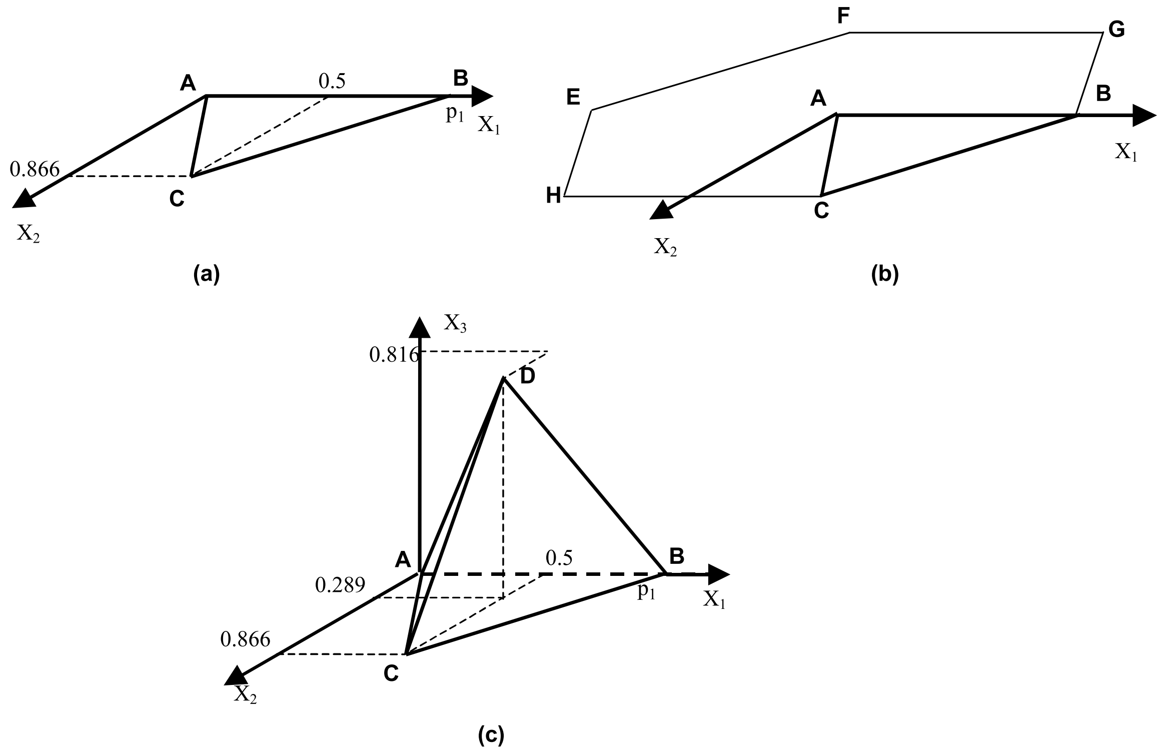

*Figure 2. Principle for building a two-factor (A and B) or a three-factor (C) Doehlert matrix.*

*Table 4. Experimental matrix with three variables.*

| | Pressure[a] | | Volume flow[b] | | Temperature[c] | | Dry matter[d] (%) | | Productivity[e] ($kg\,h^{-1}$) | |
|---|---|---|---|---|---|---|---|---|---|---|
| Nb | $p$ (hPa) | $X_1$ | $q_v$ ($l\,h^{-1}$) | $X_2$ | $T$(°C) | $X_3$ | $Y_{1e}$ | $Y_{1c}$ | $Y_{2e}$ | $Y_{2c}$ |
| 1 | 150 | 0 | 7 | 0 | 55 | 0 | 2.41 | | 0.1023 | |
| 1 | 150 | 0 | 7 | 0 | 55 | 0 | 2.61 | | 0.1053 | |
| 1 | 150 | 0 | 7 | 0 | 55 | 0 | 2.54 | | 0.1049 | |
| 1 | 150 | 0 | 7 | 0 | 55 | 0 | 2.64 | | 0.1072 | |
| **1**[f] | **150** | **0** | **7** | **0** | **55** | **0** | **2.55** | **2.55** | **0.1049** | **0.1049** |
| 2 | 179 | 1 | 7 | 0 | 55 | 0 | 2.30 | 2.21 | 0.1047 | 0.1041 |
| 3 | 164 | 0.5 | 8.5 | 0.866 | 55 | 0 | 2.17 | 1.81 | 0.1249 | 0.1260 |
| 4 | 164 | 0.5 | 7.5 | 0.289 | 70 | 0.816 | 4.25 | 4.70 | 0.1129 | 0.1122 |
| 5 | 121 | −1 | 7 | 0 | 55 | 0 | 3.11 | 3.19 | 0.1032 | 0.1037 |
| 6 | 136 | −0.5 | 5.5 | −0.866 | 55 | 0 | 3.42 | 3.78 | 0.0825 | 0.0813 |
| 7 | 136 | −0.5 | 6.5 | −0.289 | 40 | −0.816 | 1.86 | 1.41 | 0.0958 | 0.0965 |
| 8 | 164 | 0.5 | 5.5 | −0.866 | 55 | 0 | 2.79 | 3.12 | 0.0828 | 0.0818 |
| 9 | 164 | 0.5 | 6.5 | −0.289 | 40 | −0.816 | 1.78 | 1.53 | 0.0970 | 0.0984 |
| 10 | 150 | 0 | 8.0 | 0.577 | 40 | −0.816 | 1.81 | 2.50 | 0.1215 | 0.1194 |
| 11 | 136 | −0.5 | 8.5 | 0.866 | 55 | 0 | 2.45 | 2.12 | 0.1253 | 0.1262 |
| 12 | 136 | −0.5 | 7.5 | 0.289 | 70 | 0.816 | 5.55 | 5.80 | 0.1151 | 0.1137 |
| 13 | 150 | 0 | 6.0 | −0.577 | 70 | 0.816 | 8.46 | 7.77 | 0.0883 | 0.0904 |

[a] $p = 150 + 29X_1$.
[b] $q_v = 7 + 1.73X_2$.
[c] $T = 55 + 18.4X_3$.
[d] $Y_{1e}$ is the experimental DM and $Y_{1c}$ is the DM calculated according to equation (1).
[e] $Y_{2e}$ is the experimental productivity and $Y_{2c}$ is the productivity calculated according to equation (2).
[f] Average of four experiments.

Variance analysis, performed with JMP software, shows that the mean of deviation is often more important than the value of a parameter, and to exclude the terms represented in italics in equations (1) and (2). Relation (1) shows the weak negative influences of pressure ($X_1$) and feed flow ($X_2$) and the strong positive influence of temperature ($X_3$) on the DM content of extract ($Y_1$, equation 1). Correction by quadratic terms is weak, except in the case of temperature, where it is largely positive on the DM content. The interaction terms are negative for pressure–temperature and strongly negative for temperature–flow on the DM content (equation 1). Besides, volume flow ($X_2$) has a great influence on productivity, whereas temperature and pressure have a negligible one ($Y_2$, equation 2). The correction by quadratic and interaction terms does not significantly modify the trends.

*Four-factor matrix*

Our industrial partner did not want to use a continuous solid–liquid extraction in his company, so it also seemed important to study the weight fraction of the initial extract as this parameter may vary during the successive batch operations. The eight new points of the four-variable matrix (table 5) were added to the 13 previous points of the experiments (table 4) and, with this new system, $X_4$ was the coded unit value of the weight content of the feed extract ($w = 1.77 + 2.0X_4$).

The matrix calculation gives the estimated patterns represented by equation (3) for dry matter content and equation (4) for productivity (terms in italic are not significant):

$$\begin{aligned} Y_1 = {} & 2.55 - 0.48X_1 - 0.78X_2 + 2.41X_3 + 3.48X_4 \\ & + \mathit{0.15X_1^2} + \mathit{0.16X_2^2} + 2.03X_3^2 + 0.41X_4^2 \\ & + \mathit{0.20X_1X_2} - 0.82X_1X_3 - 0.32X_1X_4 \\ & - 2.51X_2X_3 + \mathit{0.13X_2X_4} + 2.58X_3X_4 \end{aligned} \qquad (3)$$

$$\begin{aligned} Y_2 = {} & 0.1049 + 0.0089X_1 + 0.0219X_2 - \mathit{0.0034X_3} \\ & + 0.1033X_4 - \mathit{0.0010X_1^2} - \mathit{0.0011X_2^2} + \mathit{0.0008X_3^2} \\ & + 0.0139X_4^2 - \mathit{0.0004X_1X_2} - \mathit{0.0019X_1X_3} \\ & - 0.0485X_1X_4 + \mathit{0.0004X_2X_3} + 0.0646X_2X_4 \\ & + 0.0188X_3X_4. \end{aligned} \qquad (4)$$

These results confirm the previously defined general trends and allow one to show the greatly positive contribution of weight content ($X_4$) as well as its positive interaction temperature on the DM content of the concentrates (equation 3). With regard to the evaporation productivity (equation 4), weight content is naturally fundamental and exerts its influence on all parameters where this variable interfere. These effects are shown in figure 3.

The pattern was verified by performing experiments with a level of variables inside the experimental area (table 6). The checking of the design was not very good because at least one variable was badly controlled. This verification allowed one to detect a dysfunction in the evaporation unit. As a matter of fact, the temperature of the heating fluid was regulated at the exit of the evaporator (TIC, figure 1) and not at the entrance (TI4). The temperature gradient between the inlet

*Table 5. First experimental matrix with four variables.*

| | Pressure[b] | | Volume flow[c] | | Temperature[d] | | Weight fraction[e] | | Dry matter[f] (%) | | Productivity[g] ($kg\,h^{-1}$) | |
|---|---|---|---|---|---|---|---|---|---|---|---|---|
| Nb[a] | $p$ (hPa) | $X_1$ | $q_v$ ($l\,h^{-1}$) | $X_2$ | $T$(°C) | $X_3$ | $w$ (%) | $X_4$ | $Y_{1e}$ | $Y_{1c}$ | $Y_{2e}$ | $Y_{2c}$ |
| 1′ (1) | 150 | 0 | 7.0 | 0 | 55 | 0 | 1.77 | 0 | 2.41 | | 0.1022 | |
| 1′ (1) | 150 | 0 | 7.0 | 0 | 55 | 0 | 1.77 | 0 | 2.61 | | 0.1053 | |
| 1′ (1) | 150 | 0 | 7.0 | 0 | 55 | 0 | 1.77 | 0 | 2.64 | | 0.1072 | |
| 1′ (1) | 150 | 0 | 7.0 | 0 | 55 | 0 | 1.77 | 0 | 2.54 | | 0.1049 | |
| **1′ (1)[h]** | **150** | **0** | **7.0** | **0** | **55** | **0** | **1.77** | **0** | **2.55** | **2.55** | **0.1049** | **0.1049** |
| 2′ (2) | 179 | 1 | 7.0 | 0 | 55 | 0 | 1.77 | 0 | 2.30 | 2.22 | 0.1047 | 0.1128 |
| 3′ (3) | 164 | 0.5 | 8.5 | 0.866 | 55 | 0 | 1.77 | 0 | 2.17 | 1.88 | 0.1249 | 0.1271 |
| 4′ (4) | 164 | 0.5 | 7.5 | 0.289 | 70 | 0.816 | 1.77 | 0 | 4.25 | 4.56 | 0.1129 | 0.1124 |
| 5′ | 164 | 0.5 | 7.5 | 0.289 | 59 | 0.204 | 3.37 | 0.791 | 5.79 | 5.84 | 0.2132 | 0.2035 |
| 6′ (5) | 121 | −1.0 | 7.0 | 0 | 55 | 0 | 1.77 | 0 | 3.11 | 3.18 | 0.1032 | 0.0950 |
| 7′ (6) | 136 | −0.5 | 5.5 | −0.866 | 55 | 0 | 1.77 | 0 | 3.42 | 3.71 | 0.0825 | 0.0802 |
| 8′ (7) | 136 | −0.5 | 6.5 | −0.289 | 40 | −0.816 | 1.77 | 0 | 1.86 | 1.55 | 0.0958 | 0.0963 |
| 9′ | 136 | −0.5 | 6.5 | −0.289 | 51 | −0.204 | 0.19 | −0.791 | 0.33 | 0.28 | 0.0102 | 0.0199 |
| 10′ (8) | 164 | 0.5 | 5.5 | −0.866 | 55 | 0 | 1.77 | 0 | 2.79 | 3.06 | 0.0828 | 0.0895 |
| 11′ (9) | 164 | 0.5 | 6.5 | −0.289 | 40 | −0.816 | 1.77 | 0 | 1.78 | 1.68 | 0.0970 | 0.1069 |
| 12′ | 164 | 0.5 | 6.5 | −0.289 | 51 | −0.204 | 0.19 | −0.791 | 0.25 | 0.16 | 0.0924 | 0.0677 |
| 13′ (11) | 136 | −0.5 | 8.5 | 0.866 | 55 | 0 | 1.77 | 0 | 2.45 | 2.18 | 0.1253 | 0.1185 |
| 14′ (10) | 150 | 0 | 8.0 | 0.577 | 40 | −0.816 | 1.77 | 0 | 1.81 | 2.72 | 0.1215 | 0.1203 |
| 15′ | 150 | 0 | 8.0 | 0.577 | 51 | −0.204 | 0.19 | −0.791 | 0.25 | (-0.10) | 0.0129 | 0.0184 |
| 16′ (12) | 136 | −0.5 | 7.5 | 0.289 | 70 | 0.816 | 1.77 | 0 | 5.55 | 5.65 | 0.1151 | 0.1051 |
| 17′ (13) | 150 | 0 | 6.0 | −0.577 | 70 | 0.816 | 1.77 | 0 | 8.46 | 7.55 | 0.0883 | 0.0895 |
| 18′ | 150 | 0 | 7.0 | 0 | 66 | 0.612 | 0.19 | −0.791 | 0.54 | 1.04 | 0.0116 | 0.0210 |
| 19′ | 136 | −0.5 | 7.5 | 0.289 | 59 | 0.204 | 3.37 | 0.791 | 6.59 | 6.68 | 0.2087 | 0.2334 |
| 20′ | 150 | 0 | 6.0 | −0.577 | 59 | 0.204 | 3.37 | 0.791 | 6.94 | 7.29 | 0.1607 | 0.1551 |
| 21′ | 150 | 0 | 7.0 | 0 | 44 | −0.612 | 3.37 | 0.791 | 4.09 | 3.60 | 0.1980 | 0.1886 |

[a] The number in parentheses is that shown in table 4.
[b] $p = 150 + 29X_1$.
[c] $q_v = 7 + 1.73X_2$.
[d] $T = 55 + 18.4X_3$.
[e] $w = 1.77 + 2.0X_4$.
[f] $Y_{1e}$ is the experimental DM and $Y_{1c}$ is the DM calculated according to equation (3).
[g] $Y_{2e}$ is the experimental productivity and $Y_{2c}$ is the productivity calculated according to equation (4).
[h] Average of four experiments.

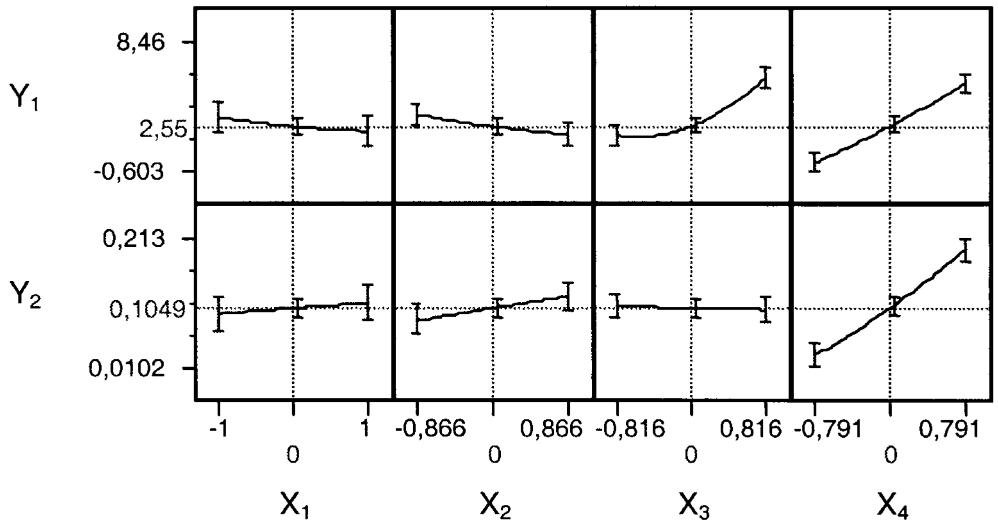

*Figure 3. Prediction profiles of influent parameter of continuous evaporation.* $Y_1$, *DM content (%);* $Y_2$, *productivity* (*kg* $h^{-1}$); $X_1$, *pressure;* $X_2$, *volume flow;* $X_3$, *temperature;* $X_4$, *weight fraction, all in coded units.*

and outlet oil depends on the amount of solvent evaporated, which itself depends on absolute pressure and feed flow, a significant error (sometimes several degrees) in the actual temperature of the oil at the entrance of the evaporator may result from this. The unit was then modified by adding a new temperature probe to the inlet pipe (TI4) and by controlling the temperature at this level.

*Table 6. Checking of the first experimental matrix with four variables.*

| | Pressure[a] | | Volume flow[b] | | Temperature[c] | | Weight fraction[d] | | Dry matter[e] (%) | | Productivity[f] ($kg\,h^{-1}$) | |
|---|---|---|---|---|---|---|---|---|---|---|---|---|
| Nb | $p$ (hPa) | $X_1$ | $q_v$ ($l\,h^{-1}$) | $X_2$ | $T$(°C) | $X_3$ | $w$ (%) | $X_4$ | $Y_{1e}$ | $Y_{1c}$ | $Y_{2e}$ | $Y_{2c}$ |
| A | 150 | 0 | 6.0 | −0.578 | 70 | 0.82 | 1.78 | 0 | 8.57 | 7.58 | 0.0813 | 0.0894 |
| B | 164 | 0.483 | 6.5 | −0.289 | 40 | −0.82 | 3.39 | 0.791 | 3.40 | 2.87 | 0.1836 | 0.1517 |

[a] $p = 150 + 29X_1$.
[b] $q_v = 7 + 1.73X_2$.
[c] $T = 55 + 18.4X_3$.
[d] $w = 1.77 + 2.0X_4$.
[e] $Y_{1e}$ is the experimental DM and $Y_{1c}$ is the DM calculated according to equation (3).
[f] $Y_{2e}$ is the experimental productivity and $Y_{2c}$ is the productivity calculated according to equation (4).

*Second four-factor matrix*

After modifying the unit, the new four-factor matrix of table 7 was performed by slightly shifting the size of the experimental area according to the equations indicated below table 7. The results shown are the average of two experiments, except for the first and the 17th, for which, respectively, six and four experiments were carried out. The matrix calculation gives the estimated pattern represented by the following equations, equation (5) for dry matter content and equation (6) for productivity (terms in italics are not significant):

$$\begin{aligned} Y_1 = {} & 3.12 - \mathit{0.81}X_1 - 2.17X_2 + 3.30X_3 + 2.88X_4 \\ & + 0.41X_1^2 + 1.54X_2^2 + 2.64X_3^2 - 0.60X_4^2 \\ & + 1.18X_1X_2 - 1.09X_1X_3 - 0.75X_1X_4 - 4.22X_2X_3 \\ & + \mathit{0.01}X_2X_4 + 2.05X_3X_4 \end{aligned} \quad (5)$$

$$\begin{aligned} Y_2 = {} & 0.1054 - 0.0012X_1 + 0.0353X_2 + 0.0028X_3 \\ & + 0.0884X_4 - \mathit{0.0004}X_1^2 - 0.0029X_2^2 + \mathit{0.0012}X_3^2 \\ & + \mathit{0.0008}X_4^2 - \mathit{0.0010}X_1X_2 - \mathit{0.0025}X_1X_3 \\ & - \mathit{0.0017}X_1X_4 - \mathit{0.0020}X_2X_3 + 0.0248X_2X_4 \\ & + \mathit{0.0026}X_3X_4. \end{aligned} \quad (6)$$

The prediction profilers of the influential parameters (not shown) are similar to those obtained with previous models (figure 3). The experimental DM contents ($Y_{1e}$) were compared with the calculated DM contents ($Y_{1c}$) obtained from equation (5) and the sums of the quadratics of the differences between the yields of each experiment [$(Y_{1e} - Y_{1c})^2$] were sometimes important. This resulted particularly from experimental errors made on DM determination. When the experiment was actually carried out in conditions that can induce high DM contents, a persistent sediment layer also stuck on the jacket of the evaporator and onto the cold thermal exchanger HE2. This deposit disturbed the results of the experiment and could disturb those of the following experiment. However, the experimental and calculated productivities ($Y_{2e}$) and ($Y_{2c}$) were generally in agreement. These results are shown in figures 4 and 5, which show the charts of actual versus predicted responses, respectively, for the DM content of the concentrate and the productivity of the process.

Residual variances ($\sigma^2$, obtained by the division of the sum of $\Sigma(y_e - y_c)^2$ by degree of freedom (48 experiments − 15 studied parameters = 33) give, respectively, 0.728 (for $Y_1$) and $12 \times 10^{-6}$ (for $Y_2$). The variance calculation allows one to exclude some insignificant parameters indicated in italics in equations (5) and (6).

This model was verified by checking some experiments with the values of parameters taken inside the experimental area (table 8) except for the experiment E, which was slightly outside the experimental area. The results are accurate considering that errors made on weight and DM measurements were inferior by 5%. Moreover, the respective means of deviation were 0.62, 0.57 and 0.91 for experiments C, D and E, and these values cover the experimental results.

*Model-building of the operation*

The purpose of this modelling is to find for a given feed weight fraction obtained after leaching, the conditions that lead to obtain (1) the best productivity of evaporation ($Y_2$) and (2) a concentrate with an 8% DM content, which is the precise concentration needed for the next step of the operation. These aims could be reached for a settled feed weight fraction of extract imposed by the previous leaching ($X_4 = 0.79$, for example) by performing a theoretical simplex on $X_1$, $X_2$ and $X_3$ variables. This methodology [4–6] can be used to obtain the optimal conditions of a process from experimental and calculated values. The coordinates ($X_1$, $X_2$ and $X_3$) of the starting simplex are given from the best response $Y_2$ in table 7 ($Y_2 = 0.1924$ for experiment 19″). The other points of the tetrahedron were obtained while using steps of −0.4, 0.6 and 0.4, respectively, for pressure, volume flow and temperature. The theoretic productivity was calculated according to equation (6). When the boundaries of the area were exceeded, the variables were then fixed to the frontier values of the Doehlert matrix, i.e. $X_1 = \pm 1$, $X_2 = \pm 0.866$ and $X_3 = \pm 0.816$. The development of the simplex (not shown) shows that the best productivity was obtained when $X_2$ and $X_3$ were at high levels and when $X_1$ was at a low level. The same conclusion was obtained for other values of weight fraction and this is in accordance with equation (6).

By replacing the expected weight fraction for evaporation ($Y_1 = 8\%$, equation 5) by the best values found for less influent variables ($X_1 = -1$ and $X_3 = 0.816$), this resolution allows one to obtain a relation between the feed flow ($X_2$) and the weight fraction of the feed solution ($X_4$),

*Table 7. Second experimental matrix with four variables.*

| | Pressure[a] | | Volume flow[b] | | Temperature[c] | | Weight fraction[d] | | Dry matter (%) | | | | Productivity ($kg\,h^{-1}$) | |
|---|---|---|---|---|---|---|---|---|---|---|---|---|---|---|
| Nb | $P$ (h Pa) | $X_1$ | $q_v$ ($l\,h^{-1}$) | $X_2$ | $T$(°C) | $X_3$ | $w$ (%) | $X_4$ | $Y_{1e}$[e] | $Y_{1c}$[e] | $\Sigma(Y_{1e}-Y_{1c})^2$[g] | $Y_{1c}$[f] | $Y_{2c}$[f] | $\Sigma(Y_{1e}-Y_{1c})^2$[h] $\times 10^6$ |
| 1″ i | 150 | 0 | 7.0 | 0 | 57.7 | 0 | 1.84 | 0 | 3.12 | 3.12 | 0.102 | 0.1054 | 0.1054 | 29 |
| 2″ | 179 | 1 | 7.0 | 0 | 57.7 | 0 | 1.84 | 0 | 2.90 | 2.72 | 0.069 | 0.1051 | 0.1038 | 4 |
| 3″ | 164 | 0.5 | 9.0 | 0.87 | 57.7 | 0 | 1.84 | 0 | 2.65 | 2.61 | 0.003 | 0.1319 | 0.1327 | 8 |
| 4″ | 164 | 0.5 | 7.7 | 0.29 | 70.9 | 0.82 | 1.84 | 0 | 5.13 | 5.50 | 0.281 | 0.1163 | 0.1161 | 43 |
| 5″ | 164 | 0.5 | 7.7 | 0.29 | 60.9 | 0.20 | 3.04 | 0.79 | 5.01 | 4.84 | 0.058 | 0.1897 | 0.1906 | 10 |
| 6″ | 121 | −1 | 7.0 | 0 | 57.7 | 0 | 1.84 | 0 | 4.16 | 4.34 | 0.068 | 0.1049 | 0.1062 | 7 |
| 7″ | 136 | −0.5 | 5.0 | −0.87 | 57.7 | 0 | 1.84 | 0 | 7.16 | 7.20 | 0.096 | 0.0735 | 0.0727 | 7 |
| 8″ | 136 | −0.5 | 6.3 | −0.29 | 44.4 | −0.82 | 1.84 | 0 | 2.56 | 2.18 | 0.285 | 0.0921 | 0.0923 | 0 |
| 9″ | 136 | −0.5 | 6.3 | −0.29 | 54.4 | −0.20 | 0.64 | −0.79 | 0.85 | 1.02 | 0.061 | 0.0314 | 0.0306 | 1 |
| 10″ | 164 | 0.5 | 5.0 | −0.87 | 57.7 | 0 | 1.84 | 0 | 4.65 | 5.36 | 1.011 | 0.0723 | 0.0724 | 1 |
| 11″ | 164 | 0.5 | 6.3 | −0.29 | 44.4 | −0.82 | 1.84 | 0 | 2.29 | 1.92 | 0.269 | 0.0908 | 0.0935 | 16 |
| 12″ | 164 | 0.5 | 6.3 | −0.29 | 54.4 | −0.20 | 0.64 | −0.79 | 0.85 | 0.67 | 0.062 | 0.0329 | 0.0314 | 10 |
| 13″ | 136 | −0.5 | 9.0 | 0.87 | 57.7 | 0 | 1.84 | 0 | 3.10 | 2.39 | 1.012 | 0.1348 | 0.1347 | 46 |
| 14″ | 150 | 0 | 8.3 | 0.58 | 44.4 | −0.82 | 1.84 | 0 | 1.96 | 3.46 | 11.436 | 0.1263 | 0.1243 | 2 |
| 15″ | 150 | 0 | 8.3 | 0.58 | 54.4 | −0.20 | 0.64 | −0.79 | 0.73 | 0 | 1.052 | 0.0428 | 0.0442 | 4 |
| 16″ | 136 | −0.5 | 7.7 | 0.29 | 70.9 | 0.82 | 1.84 | 0 | 6.50 | 6.87 | 0.342 | 0.1223 | 0.1196 | 15 |
| 17″ j | 150 | 0 | 5.7 | −0.58 | 70.9 | 0.82 | 1.84 | 0 | 12.88 | 11.38 | 4.268 | 0.0860 | 0.0881 | 116 |
| 18″ | 150 | 0 | 7.0 | 0 | 67.6 | 0.61 | 0.64 | −0.79 | 1.74 | 2.49 | 1.138 | 0.0362 | 0.0370 | 1 |
| 19″ | 136 | −0.5 | 7.7 | 0.29 | 60.9 | 0.20 | 3.04 | 0.79 | 5.94 | 6.11 | 0.064 | 0.1924 | 0.1939 | 13 |
| 20″ | 150 | 0 | 5.7 | −0.58 | 60.9 | 0.20 | 3.04 | 0.79 | 7.63 | 8.38 | 1.154 | 0.1458 | 0.1443 | 8 |
| 21″ | 150 | 0 | 7.0 | 0 | 47.8 | −0.61 | 3.04 | 0.79 | 3.74 | 2.98 | 1.194 | 0.1740 | 0.1732 | 48 |
| Sum | | | | | | | | | | | 24.025 | | | 389 |

[a] $p = 150 + 29X_1$.
[b] $q_v = 7 + 2.31X_2$.
[c] $T = 57.7 + 16.2X_3$.
[d] $w = 1.84 + 1.52X_4$.
[e] $Y_{1e}$ is the experimental DM and $Y_{1c}$ is the DM calculated according to equation (5).
[f] $Y_{2e}$ is the experimental productivity and $Y_{2c}$ is the productivity calculated according to equation (6).
[g] Sum of residues obtained in every experiment $[\Sigma(Y_{1e} - Y_{1c})^2]$.
[h] Sum of residues obtained in every experiment $[\Sigma(Y_{2e} - Y_{2c})^2]$.
[i] Average of six experiments. Only two experiments were performed with the others. The averages are shown.
[j] Average of four experiments.

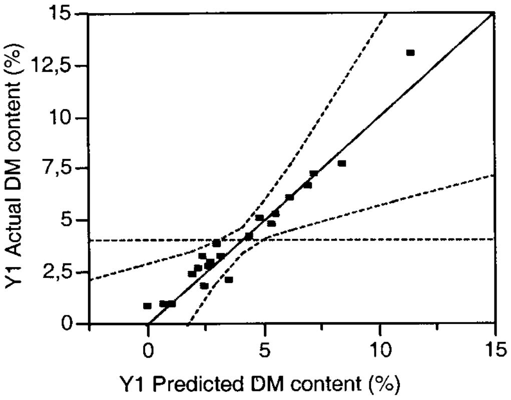

*Figure 4. Representation of the experimental versus the calculated dry matter content concentrate (see table 7).*

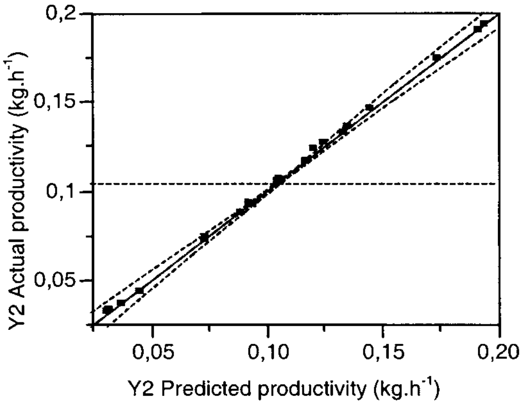

*Figure 5. Representation of the experimental versus the calculated productivity concentrate (see table 7).*

which then gives an 8% DM concentrate with the best productivity of evaporation, whatever the content of the influent extract. This conversion leads to relation (7) (figure 6), where the target area (8% DM) is specified in heavy dashes:

$$1.71 + 1.56X_2^2 - 0.62X_4^2 - 6.85X_2 + 5.15X_4 + 0.03X_2X_4 = 0. \quad (7)$$

This model was checked with two feed extracts with respective solid contents of 2.83% ($X_4 = 0.65$) and 1.38% ($X_4 = -0.30$). The resolution of equation (7) leads to respective volume flows of $9.0\,l\,h^{-1}$ ($X_2 = 0.878$) and $7.0\,l\,h^{-1}$ ($X_2 = 0$), which are the conditions to obtain an 8% DM concentrate with the best productivity. The experimental results (table 9) are in accordance with the calculated values, with errors less than 5%. The evolution of the productivity versus vol-

*Table 8. Checking of the second experimental matrix with four variables.*

| | Pressure[a] | | Volume flow[b] | | Temperature[c] | | Weight fraction[d] | | Dry matter (%) | | Productivity ($kg\,h^{-1}$) | |
|---|---|---|---|---|---|---|---|---|---|---|---|---|
| Nb | $P$ (hPa) | $X_1$ | $q_v$ ($l\,h^{-1}$) | $X_2$ | $T$ (°C) | $X_3$ | $w$ (%) | $X_4$ | $Y_{1e}$ | $Y_{1c}^{e}$ | $Y_{2e}$ | $Y_{2c}^{f}$ |
| C | 143 | −0.24 | 8.0 | 0.433 | 69.1 | 0.704 | 1.70 | −0.092 | 4.63 | 4.68 | 0.1181 | 0.1136 |
| D | 145 | −0.17 | 6.5 | −0.216 | 69.2 | 0.710 | 1.70 | −0.092 | 8.26 | 7.93 | 0.0943 | 0.0932 |
| E | 147 | −0.10 | 8.0 | −0.433 | 70.5 | 0.790 | 2.65 | 0.533 | 7.80 | 7.67 | 0.1846 | 0.1770 |

[a] $p = 150 + 29X_1$.
[b] $q_v = 7 + 2.31X_2$.
[c] $T = 57.7 + 16.2X_3$.
[d] $w = 1.84 + 1.52X_4$.
[e] $Y_{1e}$ is the experimental DM and $Y_{1c}$ is the DM calculated according to equation (5).
[f] $Y_{2e}$ is the experimental productivity and $Y_{2c}$ is the productivity calculated according to equation (6).

*Table 9. Checking of model building.*

| | Enforced variable[d] | | Optimal levels | | | | Volume flow[b] | | Dry matter (%) | | Productivity ($kg\,h^{-1}$) | |
|---|---|---|---|---|---|---|---|---|---|---|---|---|
| Nb | $w$ (%) | $X_4$ | $p$ (hPa)[a] | $X_1$ | $T$ (°C)[c] | $X_3$ | $q_v$ ($l\,h^{-1}$) | $X_2$ | $Y_{1e}$ | $Y_{1c}^{e}$ | $Y_{2e}$ | $Y_{2c}^{f}$ |
| F | 2.83 | 0.65 | 121 | −1.00 | 70.9 | 0.815 | 8.9 | 0.82 | 7.90 | 8.33 | 0.2192 | 0.2112 |
| G | 2.83 | 0.65 | 121 | −1.00 | 71.6 | 0.860 | 9.0 | 0.87 | 8.27 | 8.40 | 0.2270 | 0.2253 |
| H | 1.38 | −0.30 | 121 | −1.00 | 70.9 | 0.815 | 7.0 | 0.04 | 7.55 | 7.79 | 0.0876 | 0.0849 |

[a] $p = 150 + 29X_1$.
[b] $q_v = 7 + 2.31X_2$.
[c] $T = 57.7 + 16.2X_3$.
[d] $w = 1.84 + 1.52X_4$.
[e] $Y_{1e}$ is the experimental DM and $Y_{1e}$ is the DM calculated according to equation (5).
[f] $Y_{2e}$ is the experimental productivity and $Y_{2c}$ is the productivity calculated according to equation (6).

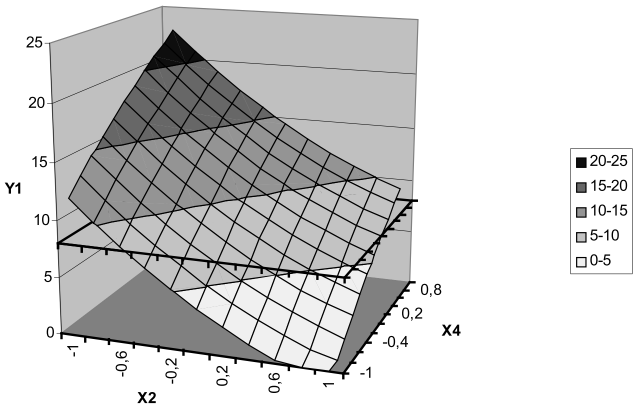

*Figure 6. Representation of the prediction of the DM content of concentrate* $Y_1$ (%) *versus the feed volume flow* ($X_2$) *and concentration* ($X_4$) *for* $X_1 = -1$ *(pressure of 121 mbar) and* $X_3 = 0.816$ *(temperature of 79.9°C).*

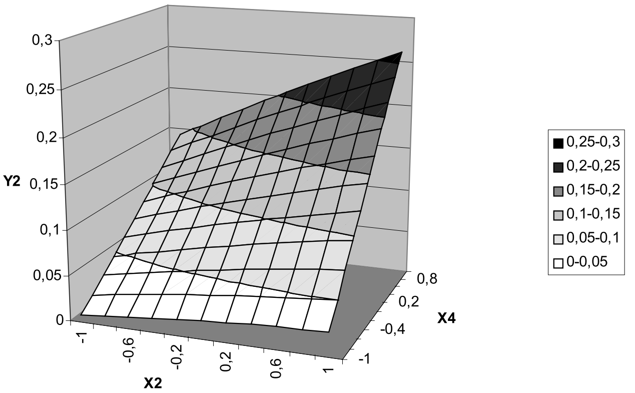

*Figure 7. Representation of the prediction of the concentrate productivity* $Y_2$ ($kg\,h^{-1}$) *versus the feed volume flow* ($X_2$) *and concentration* ($X_4$) *for* $X_1 = -1$ *(pressure of 121 mbar) and* $X_3 = 0.816$ *(temperature of* 70.9°C*).*

ume flow and weight fraction of extract in the area studied is shown in figure 7. Moreover, the variation of volume flow versus weight content of extract is shown in figure 8, where the isoresponse curves of productivity appear with an 81% DM concentrate.

### Conclusion

The use of Doehlert lattices allows the measurement of the influence of parameters while carrying out a reduced number of experiments. The lattices show the weak, negative influences of pressure and feed flow and the strong, positive effects of temperature and the weight fraction of the extract on the DM content of the concentrate. Productivity is enhanced by volume flow and particularly by the DM content. When checking the modelling, there was some dysfunction in the regulation of temperature.

After refitting the unit, the model was correctly verified with errors less than 5%. Independently of the usual experimental errors (for temperature, pressure, volume

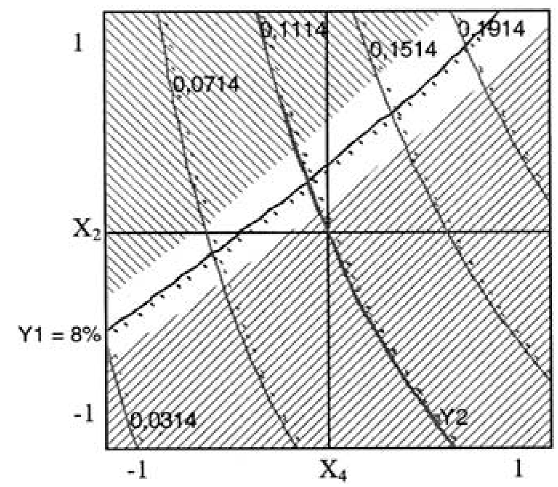

*Figure 8. Evolution of concentrate productivity in the target area for* $X_1 = -1$ *(pressure of 121 mbar) and* $X_3 = 0.816$ *(temperature of 70.9°C).*

flow, weight), which depend on the reliability of sensors and actuators, the main errors were made when determining the DM contents. In fact, the drying of vegetal products resulted from competition between the evaporation of volatile products (solvent as well as some volatile solids) and the adsorption of atmospheric water vapour. It was found that prolonged heating induced a consistent loss of solid that was proportional to a logarithmic variation of time (results not shown). These observations can be used to explain why a great difference can sometimes be observed between the experimental and the calculated values.

Finally, considering the main aim of the operation, i.e. to obtain an 8% DM concentrate with the best productivity, it was possible from an extract with a given DM content to find the conditions that allow one to reach the target, whatever the content of the solution to be evaporated.

### References

1. Doehlert, D. H., *Applied Statistics*, **19** (1970), 231.
2. Goupy, J., *Plans d'expériences pour surfaces de réponse*, (Paris: Dunod, 1999).
3. Box, G. E. P., Hunter W. G. and Hunter, J. S., *Statistics for experimenters* (New York: Wiley, 1978).
4. Spendley, W., Hext, G. R. and Himsworth, F. R., *Technometrics*, **4** (1962), 441.
5. Porte, C., Debreuille ,W. and Delacroix, A., *L'actualité Chimique* (1984), 45.
6. Porte, C., *Les Techniques de l'Ingénieur* (2002), p. 228, 1.

